# Emotional Intelligence and Professional Boredom among Nursing Personnel in Greece

**DOI:** 10.3390/jpm11080750

**Published:** 2021-07-30

**Authors:** Ioanna V. Papathanasiou, Evangelos C. Fradelos, Eleftheria Nikolaou, Konstantinos Tsaras, Lamprini Kontopoulou, Foteini Malli

**Affiliations:** 1 Nursing Department, University of Thessaly, 41500 Larissa, Greece; iopapathanasiou@uth.gr (I.V.P.); ktsa@uth.gr (K.T.); lamprini1@uth.gr (L.K.); fmalli@uth.gr (F.M.); 2 Community Nursing Lab., Nursing Department, University of Thessaly, 41500 Larissa, Greece; 3 Psychiatric Clinic A.Pisallidis A.Karipis Perea, 57019 Thessaloniki, Greece; ritanikolaou@gmail.com

**Keywords:** boredom, emotional intelligence, negative emotions, professional boredom, nurses

## Abstract

Occupational (professional) boredom results in low performance at work. It has been positively associated with high levels of anxiety and depression as well as premature death. However, occupational boredom has not been extensively studied among working nurses. This study aimed to investigate the relationship between emotional intelligence and occupational boredom in nurses working in both public (52.9%) and private (47.1%) health units in Greece. A cross-sectional study was conducted among a convenience sample of 189 nurses (84.7% females) with an average age of 40 years. Emotional intelligence was evaluated with the use of The Trait Emotional Intelligence Questionnaire-Short Form and Professional boredom was assessed with the use of the Boredom Proneness Scale. The majority of Nurses showed relatively high values of total Emotional Intelligence (EI), and marginally low values of overall Professional Boredom. A statistically significant negative correlation was found between the overall Professional Boredom of Nurses and the Well-being, Self-control, Emotionality and Sociability subscales of EI, as well as total EI (*p* < 0.001). Multiple linear regression analyses showed that the three dimensions of EI (Well-being, Self-Control and Emotionality) explained 39.0% of the variability of the total Professional Boredom of the working Nurses.

## 1. Introduction

Boredom in the workplace is a problem that has existed for centuries, with many detrimental consequences [1,2]. Among the factors that have been postulated to result in high levels of occupational boredom are monotonous and repetitive work [3,4], small workload and a highly automated work environment, with high levels of supervision. Professional boredom is directly related to dissatisfaction with work [5] and low performance [6,7] a phenomenon that is more intense among young people with less work experience, as compared to their older colleagues. It is believed that boredom can also stem from within a person [8,9] be caused by a general lack of interest and motivation to progress in life [8,9,10].

Boredom is regarded as a very broad and important problem. According to previous research [11,12], boredom along with curiosity have been identified as common causes of drug abuse. Boredom has also been positively associated with overconsumption of food and energy intake and eating disorders [13,14]. Occupational boredom in particular, is associated with health problems and can lead to the premature death of the workers, mainly due to cardiovascular disease [15]. High levels of occupational boredom are strongly associated with anxiety and depression [16], compulsive behavior, physical manifestation of stress, difficulty in interpersonal relationships and intense sensitivity. Bruursema et al. observed that employees who develop occupational boredom syndrome are more likely to misbehave (i.e., exhibit harmful and unpleasant behaviors that affect other people around them, destroy their work environment, avoid their work through absence or late arrival and even steal), and express negative emotions, especially anger, hostility and aggression [17].

The lack of cognitive skills of the individual to successfully perform his tasks can be an important factor leading to professional boredom [18]. Researchers have also tried to identify personality traits that make individuals more prone to work boredom. Extroversion has long been recognized as a key predisposing factor for the onset of work boredom, when they need to undertake simple repetitive tasks at work [19,20,21]. Interestingly, when work is enriched with additional stimuli, extroverts tend to perform better than introverts [22].

The likelihood of boredom seems to be reduced among those with a high need for achievement [23] but also among those with a high level of conscientiousness [24]. People with independent personalities, who are mainly motivated by internal rewards, are able to be satisfied with the activities they have to perform, even if these are commonplace and boring. Intelligence, age, education, specialization and gender have also been identified as other key factors related to boredom. For example, smart people tend to become bored more easily by taking on simple, non-challenging tasks [21,25]. Age seems to be negatively correlated with boredom, with younger people being more affected by monotonous tasks compared to older individuals [25,26]. Specialization has also been suggested as another feature that intensifies boredom [27].

In the nursing profession, boredom has not been studied as much as burnout. Boredom is usually expressed through dissatisfaction with work, due to increased workload, limitation in the application of knowledge, bad relationships with colleagues and lack of challenge or rewards [28].

Work occupies an important part of human life. In addition to securing the necessary financial resources, through work, people can pursue ambitions, goals and dreams, seek recognition for their achievements and place themselves socially. The degree to which the needs of the employee are met through work constitutes the degree of satisfaction from it. Nurses dedicate a significant part of their lives to work, thus job satisfaction is pivotal and an integral part of their life satisfaction [29,30].

Many studies have dealt with the evaluation of the satisfaction of the nursing staff and the factors that affect it. Excessive workload, demanding cyclical working shifts, non-assignment of roles, authoritarian management, poor opportunities for development, lack of solidarity with colleagues, the need for harmonious cooperation between many and different specialties of health professionals, the frequent contact of the health professional with people in need, but also the frequent exposure to the death of patients, compose particularly stressful and adverse working conditions, which affect the satisfaction of the nursing staff [31,32,33,34,35].

The relationship between personal and professional values is well established. A balance between work and personal life is achieved by those with higher emotional intelligence [36]. The development of personal values is usually guided by professional values [37]. When one has expectations for the profession, and their professional role is in accordance with the perception they acquire during practice, then they experience satisfaction [38]. The nursing profession is a care profession [39]. Its practice requires physical, mental and emotional effort to meet the needs of the patient, physical care and psychological support [40]. Professional nurses try to maintain the concept, art and practice of care as the ethical center of the nursing profession [41]. In this context, the ability of the caregiver consists of both performing the right techniques in his work, and creating the appropriate emotional atmosphere. Care relationships define the conditions of trust that allow the caregiver to accept the help offered, supporting the nurse-patient relationship or the therapeutic relationship [42]. It is argued that the quality of work provided as well as the quality of professional life is affected by the conditions of the profession and the personal characteristics of the professional [43]. Emotional intelligence (EI) is considered to be a set of skills or traits that are fundamental for nursing practice [44]. Goleman defined EI as the ability that people have to understand, evaluate and manage their emotions as well as the emotions of others [45]. EI is consisted by four attributes; self-awareness, self-regulation, social awareness, and relationship management [46]. According to Smith et al., EI plays a vital role in nursing practice and can affect various aspects of nursing such as clinical decision-making, collegial and inter-professional relationships, clinical environment and knowledge utilization [47]. Previous studies highlighting the positive effect that EI has on employee’s emotion regulation, overcoming challenges at work and managing with work-related stress [48]. While several studies also indicated that there is a positive relationship between EI, job performance and job satisfaction as well as EI reduces the turnover intention of employee’s [48,49]. In addition, EI has been found to be a protective factor for the negative emotions and Counterproductive Work Behaviors among employees [50,51]. According to a recent study among 202 Chinese nurses working with covid-19 patients EI was negative associated with negative emotion. More specific emotion regulation domain of EI was negative related to nurses’ depression [52]. While within the context of another study among 188 female nurses in Poland which aimed to examine the relationship of EI, nurses’ burnout and negative emotions had similar results. According to the researchers’ result there is a statistical negative relation of EI and nurses’ burnout, nurses’ trait anger, exhaustion, disengagement and sadness [53]. Despite the fact that there are papers reporting the positive effect that EI can have on boredom [54]; the empirical studies supporting this notion are limited. According to a cross-sectional study in which 184 employees from several type of industries participated there is a negative relationship between EI and professional boredom [48].

Even though both concepts boredom and EI have been fully investigated in several professions including nursing, the researches examine the relationship of those two concepts are limited. To the best of our knowledge, there are not studies investigating the relationship between boredom and EI among nursing personnel specially in Greece. Thus, the object of the present study was to investigate the Emotional Intelligence and Professional Boredom among working Nurses, the relationship between them as well as their relationship with the individual characteristics of Nurses.

## 2. Materials and Methods

### 2.1. Study Design and Participants

A cross-sectional study consisting of 189 working Nurses in Health Units, either of the public or the private sector. The inclusion criteria were: (i) be nurses or assistant nurses with at least one year working experience. and (ii) the acceptance of participation in the research. The sampling method used was Convenience Sampling.

### 2.2. Research Tools

The collection of empirical research material was carried out using a special and fully structured questionnaire, which consisted of three following parts:

- Form of personal information. It included questions about the socio-demographic and occupational characteristics of the Nurses (e.g., gender, age, marital status, number of children, level of education, postgraduate studies, work experience, sector of employment).
- Trait Emotional Intelligence Questionnaire-Short Form (TEIQue-SF). The “Trait Emotional Intelligence Questionnaire (Short Form)” in its Greek version, as validated by Petrides & Furnhman [55,56], was used to assess the degree of Nurses’ Emotional Intelligence. The TEIQ Scale consists of a total of 30 statements that assess a person’s Emotional Intelligence through a 7-point Likert scale, with a rating from “Strongly Disagree = 1” to “Strongly Agree = 7”. In 15 statements the grading is done in reverse. The TEIQ Scale according to its weighting gives 4 Sub-scales-Factors, i.e., Dimensions of Emotional Intelligence, the following: (a) “Well Being”, (b) “Self Control”, (c) “Emotionality” And (d) “Sociability”. The score of the Total Scale and the Sub-Scales results from the sum of the answers of the individual statements that make them divided by the number of them (average score per statement). Higher score values, both on the Overall Scale and the Sub-Scale, indicate higher levels of Emotional Intelligence of the respondent. The TEΙQue-SF exhibited good reliability. More specific Cronbach a for the sub-scale Well Being was found to be 0.72, for Self-Control 0.58, Emotionality 0.52, Sociability 0.61 and for the total scale was 0.85.
- Boredom Proneness Scale (BPS). The “Boredom Proneness Scale” by Farmer & Sundberg [57] was used to assess the degree of Occupational Boredom of Nurses. The BPS Scale consists of a total of 28 statements that evaluate the Professional Boredom of the employee through a 7-point Likert scale, with a rating from “Strongly Disagree = 1” to “Strongly Agree = 7”. In 9 statements the grading is done in reverse. The score of the Overall Scale results from the sum of the answers of the individual statements that make it divided by the number of them (average score per statement). Higher score values of the Overall Scale indicate higher levels of Professional Boredom of the respondent. Cronbach a for Boredom Proneness Scale was found to be 0.81 fact that supporting the excellent reliability of the scale.

### 2.3. Data Collection

Data collection was carried out in Health Units, in two cities in Greece: Thessaloniki (General Hospital of Thessaloniki “Hippocrates”, 424 General Military Training Hospital of Thessaloniki, Psychiatric structure “Alkyonis” of the Psychiatric Hospital of Thessaloniki and General Hospital of the General Hospital Pissalidi A. Karipi Thessaloniki, Clinic Medical Inter-Balkan Thessaloniki, Clinic “Agios Loukas” Thessaloniki, Boarding School “Axios” Thessaloniki) and Larissa (Clinic E. Patsidis Larissa) between January and March 2018. The participants were informed of the purpose and the objectives of the research before taking part in the study. Their participation was voluntary, anonymous and all the rules of ethics of the research were ensured, in accordance with the guidelines laid down in the Declaration of Helsinki.

### 2.4. Data Statistical Analysis

Descriptive analysis included the frequency distribution for the qualitative variables (absolute and relative % frequency) as well as estimates of the position and dispersion parameters for the quantitative variables (mean, constant deviation, median, minimum and maximum value). Inductive analysis was performed to investigate possible correlations and included t-test for independent samples, one-way analysis of variance (one-way ANOVA) for independent samples (LSD criterion was used for multiple comparisons) and its correlation coefficient Pearson (r). For the extraction of predictors, the linear regression model was applied, calculating the regression coefficient b as a measure of the relationship. The scores of the Emotional Intelligence Scale and the Occupational Boredom Scale were used as outcome measures of the studied relationships. The levels of significance (*p* value) were bilateral and the level of acceptable statistical significance was set at *p* < 5%. The processing and statistical analysis was done using the software package “SPSS (Statistical Package for the Social Science) 22.0 for Windows”.

## 3. Results

### 3.1. Summary of Demographic and Professional Characteristics

The socio-demographic and occupational characteristics of the sample are presented in Table 1. Most of the participants were females (84.7%). The participants’ age ranged from 21 to 61 years (mean value: 40.31 ± 8.95 years), with an average length of service of 13 years. Regarding their marital status, 33.9% were unmarried, 57.7% were married and 8.4% were divorced/widowed. Approximately half of them (54.0%) had 1 to 2 children. Six out of ten participants (59.8%) had tertiary education.

### 3.2. Trait Emotional Intelligence Questionnaire-Short Form

The internal consistency reliability of the Emotional Intelligence Scale (TEIQue-SF), as determined by the Cronbach’s Alpha coefficient, was found for the overall Scale to be α = 0.85, while in the four sub-Scales: α = 0.72 for Well-being, α = 0.58 for Self-Control, α = 0.52 for Emotionality and α = 0.61 for Sociability. A value of Cronbach’s Alpha > 0.70 indicates very good the reliability of internal consistency of the questions of a scale. In this case, the reliability of the Emotional Intelligence Scale is characterized by moderate to very good (Table 2).

The score for total Emotional Intelligence ranged from 3.10 to 6.57 with a mean value of 4.93 (constant deviation = 0.70) and a median value of 4.90. Half of the Nurses showed values above 4, which is the middle point of the response scale. This indicates that the majority of Nurses showed relatively high values of total Emotional Intelligence (Table 2).

Based on the mean and median value of the score of the dimensions of the Emotional Intelligence Scale, the highest value was displayed by Wellness and Emotionality, followed by Self-Control and finally Sociability (Table 2).

The divorced/widows had a lower mean value of the Well-Being dimension of EI compared to the unmarried (4.88 ± 0.81 vs. 5.48 ± 0.91 *p* = 0.022) or married (4.88 ± 0.81 vs. 5.39 ± 0.96; *p* = 0.039). Men had a higher mean value of EI levels on Emotionality than women (5.27 ± 0.66 vs. 5.00 ± 0.77; *p* = 0.079). Moreover, higher education graduates and postgraduate students had a higher mean value of EI levels on Emotionality compared to secondary school graduates (5.12 ± 0.73 vs. 4.92 ± 0.78; *p* = 0.086) and with non-holders of a postgraduate degree (5.44 ± 0.64 vs. 5.00 ± 0.76; *p* = 0.022) respectively. Postgraduate students were found to have a higher mean value of Sociality (or sociability?) dimension of EI than non-postgraduates (4.73 ± 0.63 vs. 4.38 ± 0.93; *p* = 0.050). Postgraduate students had a higher average overall Emotional Intelligence score than non-postgraduate students (5.22 ± 0.55 versus 4.90 ± 0.70; *p* = 0.072).

### 3.3. Boredom Proneness Scale

The Internal Consistency Reliability Scale of the Boredom Proneness Scale (BPS), determined by Cronbach’s alpha, was found to be α = 0.81, which characterizes the internal consistency reliability of the Scale questions as “very good” (Table 2).

The score for the total Boredom Proneness Scale ranged from 1.75 to 4.68 with a mean value of 3.32 (±0.67) and a median value of 3.36. The median value was slightly lower than the value 4 which is the middle point of the response measurement scale, indicating that the majority of Nurses marginally showed low values of overall Professional Boredom (Table 2).

Results from the bivariate analysis between sample characteristics, subscales of Trait Emotional (teique-SF) and Boredom Proneness Scale (BPS) are presented in Supplementary Table S1.

### 3.4. Correlation between Emotional Intelligence and Professional Boredom

Table 3 presents the investigation of the relationship between the Emotional Intelligence Scale (TEIQue-SF) and the Boredom Proneness Scale (BPS) of working Nurses.

A statistically significant negative correlation was found of each subscale of the Emotional Intelligence and the overall scale with the overall Professional Boredom of Nurses (*p* < 0.001). In particular, as the overall EI (emotional intelligence) scale and its individual 3.dimensions related to Well-Being, Self-Control, Emotionality and Sociability increased, the levels of overall Professional Boredom decreased (Table 3).

### 3.5. Determinants of Professional Boredom

Table 4 presents the multivariate investigation of the relationship, for the extraction of determinants or predictors, between the Boredom Proneness Scale (BPS) as a dependent variable and the aspects of the Emotional Intelligence Scale (TEIQue-SF) as independent variables.

The statistical model includes three dimensions of Emotional Intelligence (Well-being, Self-control, Emotionality) as determinants or predictors of the total Professional Boredom of working Nurses (F = 41.076 and *p* < 0.001).

Each dimension of Emotional Intelligence was significantly negatively associated with the overall Occupational Boredom of Nurses (*p* = 0.001). In particular, an increase in Well-Being levels by one unit causes a decrease in the levels of total Professional Boredom by 0.173 units (adjusted factor β = −0.173 with 95% confidence interval from −0.272 to −0.075). An increase of the Self-Control levels by one unit causes a decrease of the levels of the total Professional Boredom by 0.134 units (adjusted coefficient β = −0.134 with 95% confidence interval from −0.238 to −0.029), while an increase in the levels of Emotion by one unit causes a decrease in the levels of total Professional Boredom by 0.310 units (adjusted factor β = −0.310 with 95% confidence interval from −0.430 to −0.191).

The statistical model with the three dimensions of Emotional Intelligence explained 39.0% of the variability of the total Professional Boredom of the working Nurses (Adjusted R^2^ = 39.0%).

## 4. Discussion

This research investigated the relationship between Emotional Intelligence and Professional Boredom among Nurses working in health units either in public or private sector. Regarding the EI scale, the highest value was shown by Well Being and Emotionality followed by Self-Control and Sociability.

The relationship of EI with the characteristics of nurses revealed that the divorced/widows had a lower mean value of EI levels related to Well-Being when compared to the unmarried. Our findings are in accordance with previous work which showed that marital status and parental status affect the subjective well-being of nursing staff, with married people having higher levels of positive emotions and life satisfaction when compared to those who are alone who have higher levels of negative emotions [58]. The significant impact of marital status on nurses’ reported job satisfaction and stress levels has also been previously highlighted, with single people reporting higher levels of work-related stress than married people [59]. This may be due to the fact that married workers may receive emotional support from their spouse at home after work.

Self-control was not related to the characteristics of the nurses. Regarding Emotionality the present study showed its link to gender, with men having a higher mean value of the emotionality subscale of EI than women. This finding is of significant importance, since male nurses are a minority in the nursing profession and are called upon to confront cultural beliefs that women are better suited to specific emotional tasks. Nevertheless, they must meet the demands of an emotionally intense work [60]. Similar findings regarding the relationship between emotionality and gender, have been previously reported by McNulty and colleagues who conducted a study to determine differences in gender, age or culture in the scores of Emotional Intelligence traits among Radiology students in four countries. A possible explanation for the fact that men score higher on the emotionality factor than women was the likelihood that participants may have completed self-report questionnaires in line with profession-based expectations and stereotypes [61].

Emotionality was also correlated with the educational level, as it was found that higher education graduates and postgraduate students had a higher mean value of EI levels on Emotionality compared to secondary school graduates and with non-holders of a postgraduate degree respectively.

Regarding Sociality, the present study found it to be related to age. Specifically, as age increased, so did EI levels related to (r = 0.128). Sociability was also correlated with educational level as postgraduate students were found to have a higher mean value of EI levels related to than non-postgraduates. Finally, sociability was also correlated with previous service and in particular it was found that as the years of service increased, so did the EI levels related to Sociality (r = 0.133). This finding is consistent with that of Stami et al. who found that the sociability dimension of emotional intelligence was greater among those with a high level of employment and a high level of education. This result could be due to the fact that trust and experience, which are reasonably assumed to be the result of higher levels of employment and education, will enhance the sociability of individuals. In addition, teamwork and close collaboration environment within the interdisciplinary team requires sociability skills, so these will logically increase as a result of the regular and continuous employment of professionals in higher roles [62].

Finally, the total EI is statistically significant, with the possessors of a master’s degree. Specifically, postgraduate students had a higher average value of overall Emotional Intelligence levels than non-postgraduate students, in accordance with previous studies [63]. Similar to our study, registered nurses (RNs) were generally found to have higher levels of emotional intelligence than other occupations, and that nurses with a master’s degree had higher levels of emotional intelligence than those with associate degrees. This finding highlights the importance of continuing education and training after obtaining a basic degree, which can also lead to personal enrichment.

In the study of the Professional Boredom scale, low values of total Professional Boredom were observed. Also, no statistically significant relationships were found between the characteristics of the working Nurses and the overall Professional Boredom. In contrast to our findings, Abazari et al. found that there is a significant relationship between professional boredom and educational level (i.e., those with lower education had a higher-level job boredom proneness (JBP) as well as between professional boredom and years of employment [64].

As the levels of EI related to well-being, self-control, emotionality and sociability increased and total EI, so did the levels of overall Professional Boredom. A negative relationship between professional boredom and emotional intelligence was also reported by Wan et al. [48]. Boredom in the workplace is the result of performing monotonous or boring tasks or tasks that do not attract employees, who cannot maintain their interest in the work for a long time. One explanation for this negative relationship between boredom and emotional intelligence is that people with higher levels of emotional intelligence can facilitate creativity to reduce boredom and think of new ways and approaches at work. In addition, it has been reported that emotionally intelligent people tend to be more optimistic and able to put themselves in positive emotional states and that people with high levels of emotional intelligence are able to cope with complex and demanding tasks that would otherwise lead to high stress levels. Thus, people with higher levels of emotional intelligence can approach their work in creative ways to make their work more interesting, while adopting a positive perspective and will not be easily overwhelmed by complex tasks that would otherwise discourage them [48].

Finally, in a more detailed examination of the investigation of the relationship between the Professional Boredom Scale (BPS) as a dependent variable and the dimensions of the Emotional Intelligence Scale (TEIQue-SF variables) for the extraction of determinants or predictors in the nurses of the sample, we observed that as the dimensions of wellbeing, self-control and emotionality of EI increased so did the levels of Boredom in the workplace decrease. This finding is highlighting inverse relation of EI and professional boredom. This finding is in agreement and reinforce other studies that are reporting the positive effect that EI can have in various work-related variables. According to a cross-sectional study among nurses in Nigeria EI can act as a mediator and reduce the negative effects that burnout has on nurses’ productivity. The researches attributed the effect that EI has, by showing that emotionally intelligent individuals are less likely to adopt counterproductive behaviors due to their self-control [65]. Similarly, Ranjbar Ezzatabadi et al., reported that EI does not only have positive effect on work-related variables such as job satisfaction and performance, but has also positive effect on the quality of the provided care. Indicating that emotionally intelligent individuals have better communications skills and tend to be more satisfied by their profession [66]. It is a fact that individuals that are characterized by self-control, sociality and are aware of their emotion can be respectful for other people emotions and can adopt effective communication technics leading to provide effective nursing care [67,68].

Despite the novelty of the present study and the fair sample size there are few limitations that must be mentioned. First of all, the cross-sectional study design does not allow to the researchers to have a deep insight how the measured variables change within the course of professional life. In addition, the convenience sample is a limitation to. Futures studies must be conducted employing prospective study design in order to understand the variations of EI and job boredom through time.

## 5. Conclusions

Results from the present study reinforce the general notion about the positive effect that EI has on workforce and organizations. EI has being shown to have an inverse relationship with professional boredom. According to the results of the present study emotionally intelligent individuals are less likely to experience job boredom. More specific the mentioned inverse relationship has been found in specific EI domains such as well-being, self-control and emotionality. Directors and managers of the healthcare organizations and services must be aware about the positive effect that EI can have on productivity and other work-related factors such professional boredom and implement strategies for enhancing EI of the employees.

## Figures and Tables

**Table 1:** Personal characteristics of the Nurses (*n* = 189).

Characteristics	n	%
*Sex*		
Male	29	15.3%
Female	160	84.7%
*Age* (*years*)		
mean ± st. dev.	40.31 ± 8.95	
min–max	21–61	
*Marital Status*		
Unmarried	64	33.9%
Married	109	57.7%
Divorced	15	7.9%
Widow/Widower	1	0.5%
*Number of Children*		
0	72	38.1%
1–2	102	54.0%
≥3	15	7.9%
*Education Level*		
Secondary Education	76	40.2%
Tertiary Education	104	55.0%
University Education	9	4.8%
*Postgraduate studies*		
Yes	17	9.0%
No	172	91.0%
*Work experience* (*years*)		
mean ± st. dev.	13.77 ± 8.73	
min–max	1–35	
*Nursing sector of employment*		
Medical	60	31.7%
Surgical	35	18.5%
Psychiatric	33	17.5%
Other	61	32.3%
*Health Unit*		
Public Sector	100	52.9%
Private Sector	89	47.1%

**Table 2:** Trait Emotional Intelligence Questionnaire-Short Form (TEIQue-SF) and Boredom Proneness Scale (BPS) of the Nurses (*n* = 189).

	Cronbach’s Alpha	Mean ± SD	Median	Min–Max
**TEIQue-SF**				
Well-being	0.72	5.38 ± 0.94	5.50	2.33–7.00
Self-control	0.58	4.63 ± 0.89	4.67	2.33–6.83
Emotionality	0.52	5.04 ± 0.76	5.13	3.25–6.88
Sociability	0.61	4.41 ± 0.91	4.33	2.17–7.00
Overall Scale	0.85	4.93 ± 0.70	4.90	3.10–6.57
**BP Scale**				
Overall Scale	0.81	3.32 ± 0.67	3.36	1.75–4.68

**Table 3:** Correlation between TEIQue-SF and BPS of Nurses (*n* = 189).

TEIQue-SF Scale	BPS Scale	
	Pearson (r)	*p* Value
Well being	−0.502	<0.001
Self-control	−0.475	<0.001
Emotionality	−0.552	<0.001
Sociability	−0.407	<0.001
Overall Scale	−0.652	<0.001

**Table 4:** Multiple linear regression with the Boredom Proneness Scale (BPS) as a dependent variable and the aspects of the Nurses’ Emotional Intelligence Scale (TEIQue-SF) (*n* = 189) as independent variables.

Independent Variables	β	SE	95% CI	*p* Value
a	6.431	0.284	5.870 to 6.991	<0.001
Well being	−0.173	0.050	−0.272 to −0.075	0.001
Self-control	−0.134	0.053	−0.238 to −0.029	0.012
Emotionality	−0.310	0.061	−0.430 to −0.191	<0.001

Adjusted R^2^ = 39.0%; F = 41.076; *p* < 0.001.

## Data Availability

Data are available upon reasonable request.

## Supplementary Materials

The following are available online at https://www.mdpi.com/article/10.3390/jpm11080750/s1, Table S1: Correlations between Sample Characteristics, Subscales of Trait Emotional Intelligence Questionnaire-Short Form (TEIQue-SF) and Boredom Proneness Scale (BPS).
