# Supplementary material for: Emotional Intelligence and Professional Boredom among Nursing Personnel in Greece
Source: J Pers Med. 2021 Jul 30;11(8):750. doi: 10.3390/jpm11080750 (PMC8400954; doi:10.3390/jpm11080750)
Supplement: Supplementary file 1 [file jpm-11-00750-s001.zip › jpm-1270328-supplementary.pdf]

Supplementary Table: Correlations between Sample Characteristics, Subscales of Trait Emotional Intelligence Questionnaire-Short Form (TEIQue-SF) and Boredom Proneness Scale (BPS).

Characteristics	Emotional Intelligence TEIQue-SF					Professional Boredom - BPS
	Well being	Self-control	Emotionality	Sociability	Overall Scale	
**Gender**						
Male	5.21 ± 1.15	4.56 ± 0.83	5.27 ± 0.66	4.46 ± 0.76	4.96 ± 0.65	3.32 ± 0.80
Female	5.41 ± 0.90	4.64 ± 0.91	5.00 ± 0.77	4.40 ± 0.93	4.92 ± 0.71	3.32 ± 0.65
t	1.067	0.472	1.767	0.314	0.273	0.021
P value	0.288	0.638	**0.079**	0.754	0.785	0.983
**Age (years)**						
r	-0.07	-0.068	-0.061	0.128	-0.033	0.059
P value	0.336	0.354	0.403	**0.08**	0.648	0.418
**Marrital Status**						
Unmarried (1)	5.48 ± 0.91	4.67 ± 0.89	5.12 ± 0.67	4.29 ± 0.88	4.95 ± 0.69	3.27 ± 0.72
Married (2)	5.39 ± 0.96	4.57 ± 0.89	5.03 ± 0.81	4.51 ± 0.92	4.94 ± 0.72	3.34 ± 0.65
Divorced/ Widow/Widower (3)	4.88 ± 0.81	4.86 ± 0.91	4.79 ± 0.68	4.19 ± 0.91	4.77 ± 0.56	3.30 ± 0.67
F	2.702	0.822	1.239	1.749	0.472	0.221
P value	**0.07**	0.441	0.292	0.177	0.624	0.802
Post Hoc Tests	(1)>(3) p=0.022					
	(2)>(3) p=0.039					
**Number of Children**						
r	-0.072	-0.037	-0.14	-0.003	-0.07	0.003
P value	0.325	0.613	**0.065**	0.97	0.335	0.966

**Education Level**						
Secondary Education	5.34 ± 0.91	4.53 ± 0.93	4.92 ± 0.78	4.32 ± 0.95	4.84 ± 0.71	3.34 ± 0.69
Tertiary / University Education	5.41 ± 0.96	4.70 ± 0.86	5.12 ± 0.73	4.47 ± 0.88	4.99 ± 0.68	3.30 ± 0.66
t	0.485	1.306	1.725	1.128	1.429	0.358
P value	0.628	0.193	**0.086**	0.261	0.155	0.721
**Postgraduate Studies**						
Yes	5.58 ± 0.68	4.88 ± 0.73	5.44 ± 0.64	4.73 ± 0.63	5.22 ± 0.55	3.21 ± 0.52
No	5.36 ± 0.96	4.60 ± 0.91	5.00 ± 0.76	4.38 ± 0.93	4.90 ± 0.70	3.33 ± 0.69
t	0.919	1.224	2.316	2.056	1.812	0.684
P value	0.359	0.223	**0.022**	**0.05**	**0.072**	0.495
**Work Experience (years)**						
r	-0.051	-0.071	-0.058	0.133	-0.031	0.037
P value	0.49	0.334	0.431	**0.069**	0.675	0.617
**Nursing Sector of Employment**						
Medical	5.33 ± 0.90	4.67 ± 0.89	5.08 ± 0.76	4.26 ± 0.91	4.90 ± 0.70	3.29 ± 0.64
Surgical	5.40 ± 0.95	4.54 ± 0.97	4.93 ± 0.78	4.32 ± 0.75	4.85 ± 0.72	3.36 ± 0.70
Psychiatric	5.40 ± 0.93	4.68 ± 0.92	5.16 ± 0.72	4.59 ± 0.90	5.00 ± 0.72	3.36 ± 0.64
Other	5.40 ± 1.00	4.61 ± 0.86	5.00 ± 0.76	4.52 ± 0.98	4.96 ± 0.66	3.29 ± 0.72
F	0.075	0.185	0.665	1.436	0.312	0.143
P value	0.973	0.906	0.575	0.234	0.817	0.934
**Health Unit**						
Public Sector	5.23 ± 1.03	4.63 ± 0.88	5.10 ± 0.81	4.47 ± 0.89	4.94 ± 0.73	3.25 ± 0.71
Private Sector	5.47 ± 0.83	4.63 ± 0.92	4.98 ± 0.69	4.35 ± 0.93	4.91 ± 0.66	3.39 ± 0.63
t	1.309	0.048	1.088	0.894	0.33	1.408
P value	0.192	0.962	0.278	0.373	0.742	0.161
**Data are given in mean ± sd**						
